# Orbit/CLASP Is Required for Germline Cyst Formation through Its Developmental Control of Fusomes and Ring Canals in *Drosophila* Males

**DOI:** 10.1371/journal.pone.0058220

**Published:** 2013-03-08

**Authors:** Chie Miyauchi, Daishi Kitazawa, Itaru Ando, Daisuke Hayashi, Yoshihiro H. Inoue

**Affiliations:** Insect Biomedical Research Center, Graduate School of Science and Technology, Kyoto Institute of Technology, Matsugasaki, Kyoto, Japan; Institut de Génétique et Développement de Rennes, France

## Abstract

Orbit, a *Drosophila* ortholog of microtubule plus-end enriched protein CLASP, plays an important role in many developmental processes involved in microtubule dynamics. Previous studies have shown that Orbit is required for asymmetric stem cell division and cystocyte divisions in germline cysts and for the development of microtubule networks that interconnect oocyte and nurse cells during oogenesis. Here, we examined the cellular localization of Orbit and its role in cyst formation during spermatogenesis. In male germline stem cells, distinct localization of Orbit was first observed on the spectrosome, which is a spherical precursor of the germline-specific cytoskeleton known as the fusome. In dividing stem cells and spermatogonia, Orbit was localized around centrosomes and on kinetochores and spindle microtubules. After cytokinesis, Orbit remained localized on ring canals, which are cytoplasmic bridges between the cells. Thereafter, it was found along fusomes, extending through the ring canal toward all spermatogonia in a cyst. Fusome localization of Orbit was not affected by microtubule depolymerization. Instead, our fluorescence resonance energy transfer experiments suggested that Orbit is closely associated with F-actin, which is abundantly found in fusomes. Surprisingly, F-actin depolymerization influenced neither fusome organization nor Orbit localization on the germline-specific cytoskeleton. We revealed that two conserved regions of Orbit are required for fusome localization. Using *orbit* hypomorphic mutants, we showed that the protein is required for ring canal formation and for fusome elongation mediated by the interaction of newly generated fusome plugs with the pre-existing fusome. The *orbit* mutation also disrupted ring canal clustering, which is essential for folding of the spermatogonia after cytokinesis. Orbit accumulates around centrosomes at the onset of spermatogonial mitosis and is required for the capture of one of the duplicated centrosomes onto the fusome. Moreover, Orbit is involved in the proper orientation of spindles towards fusomes during synchronous mitosis of spermatogonial cysts.

## Introduction

In many animal species, germ cells synchronously develop as a syncytium of clonally related cells known as a cyst [1], [2]. The cells within a cyst are interconnected and share a cytoplasm through intercellular bridges. Cysts originate from incomplete cytokinesis during cell division. In *Drosophila* oogenesis, 16 cystocytes are generated after four rounds of mitosis of a cystoblast derived from a germline stem cell (GSC); these 16 interconnected cells constitute a cyst. After cyst formation, a single cell within the 16-cell cyst will determine its fate as an oocyte. The remaining 15 cells in the cyst differentiate into nurse cells, which provide mRNA and proteins to the oocyte [1].

In *Drosophila* spermatogenesis, every cell within a cyst equally determines the cell’s fate, and all cells synchronously develop to generate 64 mature sperm [3], [4]. At the tip of the testis, GSCs receive the Upd signal, which allows the cells to maintain their stem cell characteristics from adjoining hub cells [5], [6]. A proximal daughter cell derived from a GSC receives the Upd signal and becomes a self-renewing GSC. The distal daughter cell then leaves the niche and begins to differentiate as a spermatogonium. The spermatogonium undergoes four cell cycles and generates 2-cell, 4-cell, 8-cell, and 16-cell cysts at the end of each mitotic division [3]. During mitosis, all spermatogonia within a cyst synchronously perform cell division. Cytokinesis in spermatogonial mitosis, and during the following meiotic divisions, terminates incompletely, and the contractile rings transform into cytoplasmic bridges called ring canals [7]. These 16 spermatocytes synchronously enter a growth phase and increase their volume. After completion of the cell growth phase, a cyst of 16 spermatocytes generates 64 spermatids, because of synchronous meiotic divisions. However, the detailed mechanism that coordinates germ cell division and controls synchronous cyst development in males remains poorly understood.

Clonally related germ cells in a cyst are interconnected through ring canals [3], [8], [9]. The sharing of cytoplasm through ring canals plays an important role in synchronizing the cell cycle and differentiation of cyst cells [10], [11], [12], [13]. Constriction of contractile rings during cytokinesis is arrested, and the midbody transforms into a cytoplasmic bridge. Formation of the ring canal is initiated during cytokinesis, with the appearance of phosphorylated tyrosine epitopes on the contractile rings [7], [9]. Many ring canal components have been identified in females, but fewer in males. In addition to F-actin, which is a major contractile ring component, orthologs of several actin-binding proteins, such as Hts and Kelch, are found in female ring canals [9], [12], [14], [15]. By contrast, in matured male ring canals, F-actin and myosin II disappear after cytokinesis, while three types of septin (Peanut, Sep1, and Sep2), anillin, and centralspindlin components such as Pav and its partner Nesd (which are contractile ring components) continue to be present [7], [16], [17].

To tether germ cells within a cyst, a germline-specific cytoskeleton known as a fusome plays an essential role [18]. The fusome initially arises from a spherical structure, called the spectrosome, in the GSC. The fusome elongates and extends clonally towards related germ cells in a cyst, through the ring canals. The fusomes are involved in orienting mitotic spindles to achieve synchronized mitoses of cystocytes [19]. The female fusome has been shown to be associated with stable microtubules and to contain ER-derived vesicles along with a meshwork of membrane skeleton proteins [20]. The membrane skeleton proteins α- and β-spectrin, ankyrin, and HtsF protein were identified as major components of the *Drosophila* female fusome [14], [21], [22], [23], and are required for the maintenance of fusome integrity. The ER-containing proteins Rtnl1, Sec61α, and protein disulfide isomerase (PDI) are also enriched in the female fusome [24], [25]. In addition to vesicle-related proteins, the female fusome contains recycling endosomal and lysosomal proteins [26], [27]. Therefore, the fusome has been proposed as a center of membrane recycling and a signal for the supply of ER-derived components to oocytes [26]. Furthermore, microtubule motors (e.g., Dhc and KLP61F) and microtubule-associated proteins (e.g., Lis1) are required to generate a normal female fusome structure [28], [29], [30]. The KLP61F protein is localized in the fusome at the interphase and shifts to the spindle microtubules at the mitotic stage [29].

Fusome composition and development differs between males and females. In the male fusome, no membrane cisternae structures have been observed [3], [31], [32]. The fusome-localized proteins, and genes required for fusome formation, are less characterized in males. Additionally, the male fusome comprises α-spectrin, HtsF, and F-actin [7], but fewer ER-related fusome materials [26]. Male GSCs possess a checkpoint, which monitors correct centrosome orientation prior to mitosis. This checkpoint ensures proper spindle orientation towards the hub cells, which in turn ensures asymmetric GSC division [33]. The spectrosome localization of cyclin A via Par-1 plays an important role in the centrosome orientation checkpoint [34]. In female fusomes, the interaction between the centrosome and fusome is important for cystocyte division in the egg chambers [13], [14]. A similar role of male fusomes has not been observed.

Previous studies have identified a *Drosophila* ortholog of CLASP, known as Orbit/Mast (hereafter referred to as Orbit), as a microtubule-associated protein required for the formation of ring canals and fusomes in oogenesis [13], [35]. Orbit exhibits a distinct localization on ring canals and the ovarian fusome in the egg chamber; however, its involvement in male germline cyst formation has not been investigated. The *orbit* gene encodes a microtubule-associated protein, which is required for proper mitotic spindle organization in early embryos, larval neuroblasts, and cultured cells [35], [36]. Orbit is required to maintain spindle bi-polarity, and it facilitates kinetochore attachment to microtubules during chromosome segregation [37], [38]. The mammalian ortholog (CLASP) is enriched at the plus ends of microtubules and contributes to the stabilization of the cell cortex at the interphase [39]. CLASP preferentially localizes near the plus ends of growing spindle microtubules and in kinetochore regions in mitosis; further, it is required for attached microtubules to exhibit normal dynamic behavior at the kinetochore [38], [40]. Previous studies of Orbit in *Drosophila* oogenesis have shown that Orbit plays a central role in the formation of ring canals and fusomes within a developing cyst [13]. Moreover, immunostaining of male meiotic cells demonstrated that the protein remained predominantly within the spindle envelope and associated with kinetochores and spindle microtubules at the metaphase [41]. When entering the anaphase, Orbit is distributed along the interior central spindle microtubules and begins to concentrate on the mid-part of the central spindle, where it remains as a cleavage furrow during telophase. Accordingly, Orbit plays an essential role in cytokinesis during male meiosis.

In the present study, we aimed to identify a novel factor that is essential for germline cyst formation and characterize its role in *Drosophila* spermatogenesis. We examined the cellular localization and role of the microtubule-associated protein Orbit in male premeiotic cysts. Distinct localization of Orbit was first observed on the spectrosome of GSCs. Subsequently, Orbit became localized on cell division apparatuses, such as centrosomes and spindle microtubules, in the dividing spermatogonia. After cytokinesis, Orbit remained localized on ring canals, and thereafter, was found on the spectrosome and fusomes in premeiotic cells at the interphase. Surprisingly, fusome localization of Orbit was not affected by microtubule depolymerization. Instead, fluorescence resonance energy transfer (FRET) experiments suggested that Orbit was closely associated with F-actin in the fusome of premeiotic spermatocyte cysts. A region distinct from the microtubule-binding domain is required for fusome localization. We propose that Orbit may play a role as an actin-binding protein during the formation of the germline-specific cytoskeleton. Genetic analyses of *orbit* mutants revealed that Orbit is required for the formation of the fusome and ring canals in spermatogenesis. Moreover, the protein tends to accumulate around centrosomes, where it is involved in capturing spindle poles onto the fusome and synchronizing the mitoses of spermatogonial cysts. Thus, we conclude that Orbit is useful for the control of synchronous spermatogonial divisions, through maintaining the interaction between the fusome and spindle poles.

## Materials and Methods

### Molecular Cloning

We constructed plasmid DNAs, namely, pUASp-GFP-Orbit and pUASp-mRFP-Orbit, for the expression of Orbit proteins containing GFP and mRFP tags in germline cells. To obtain the pUASp-GFP-Orbit plasmid, a 0.7-kb cDNA fragment for EGFP was amplified using PCR, with pEGFP-C2 (Clontech, Mountain View, CA, USA) as a template, and digested using the enzymes *Mlu*I and *Bgl*II. We also prepared a 5.2-kb *Bam*HI*/Sal*I cDNA fragment encoding the full-length *orbit* (beginning from the first ATG codon to the stop codon, including the 3′ untranslated region [UTR] sequences). The restriction fragments for EGFP and Orbit were ligated to each other, inserted into a pKF3 vector (Takara Bio, Kyoto, Japan), and treated with the enzymes *Mlu*I and *Sal*I. A 5.9-kb *Mlu*I/*Sal*I cDNA fragment, encoding the full-length Orbit fused with EGFP at the N-terminus converted into a blunt end fragment, was prepared and inserted into a *Bam*HI site of the pUASp vector after blunt-ended conversion. We confirmed the insertion of the DNA sequences into the plasmid, pUASp-EGFP-Orbit, and expressions of the fusion proteins by using western blot analysis. The pUASp-EGFP-NOrbit plasmid, which induced the expression of the N-terminal Orbit (1–627), was constructed as follows: pUASp-EGFP-Orbit DNA was digested by *Eco*RI, after which a blunt-end conversion and ligation was performed to remove a 3.1-kb *Eco*RI fragment and introduce a stop codon directly behind Asn^627^.

The pUASp-mRFP vector was constructed as follows: mRFP cDNA fragments were amplified using PCR, with pDsREd-Monomer (Clontech, Mountain View, CA, USA) as a template to avoid termination by the addition of C residues before the stop codon. The DNA fragment digested by *Kpn*I and *Not*I was inserted into the pUASp vector. This constructed plasmid, pUASp-mRFP, was used as an expression vector for full-length and truncated Orbit proteins with an RFP tag. The 5.2-kb *Bam*HI/*Xba*I cDNA fragment encoding the full-length protein (beginning from first ATG codon to the stop codon with 3′ UTR sequences) was inserted into pUASp-mRFP to express the Orbit polypeptide fused with the mRFP tag at its N-terminus, through an intermediate 10-amino acid linker. We amplified the cDNA encoding the Heat (1–252), HRI (250–900), or HRII (900–1492) regions by using PCR and inserted each fragment into the pUASp-mRFP vector to express the truncated Orbit polypeptide fused with the mRFP tag at the N-terminus. We confirmed the DNA sequences of each of the resulting plasmids and the expressions of the fusion proteins by using western blot analysis.

To construct plasmid DNA for the expression of a Venus-Orbit fusion protein in germline cells, we inserted a 5.1-kb cDNA, encoding the full-length Orbit protein, into a pPVW vector from the *Drosophila* Gateway collection (Drosophila Genomics Resource Center) in frame.

### Fly Stocks

Two previously described stocks, *y w* and *Ub*-*β-tubulin-GFP* [41], were used as wild-type controls for the cytological and time-lapse studies. The *orbit^7^* mutant was described previously [41]. We used *bam-Gal4::vp16* as the Gal4 driver for spermatocyte-specific gene induction and *nos-Gal4::vp16* for gene induction of GSCs to spermatocytes. The *UASp-β-actin-mRFP* and *UAS-ECFP-β-actin* stocks were obtained from the Bloomington Stock Center (Indiana University, Bloomington, IN, USA). To obtain transgenic lines, the plasmid DNA described above was microinjected into early embryos from *y w* by using a helper P-element plasmid. We established several *w^+^* transgenic lines for each transgene and performed genetic crosses to determine the insertional chromosomes. To observe fluorescently tagged Orbit, we established fly stocks carrying *UASp* transgenes and *bam-Gal4::vp16* (for observation of 8-cell cysts to meiocytes), and the *nos-Gal4::vp16* insertion (for observation of GSCs to 4-cell cysts), in the homozygous state.

### Rescue Experiments

To confirm the functionality of Orbit fused with GFP, mRFP, or Venus fluorescence tags, we generated homozygous *orbit^7^* males carrying the *UASp* transgene and *nos-Gal4::vp16 to* induce the expression of fusion proteins in GSCs to spermatogonia. We examined the fertility of single males raised at 25°C by crossing with *Canton S* females. Every 10 single males with germline cells expressing each of the three fusion proteins showed complete restoration of the male sterile phenotype of *orbit^7^* males. Thus, Orbit fused with GFP, mRFP, or Venus fluorescence tags is functional.

### Drug Treatment of Testis Cells

#### Colchicine

Testes from young adult males were dissected, and the spermatocyte cysts were spread on a cover glass. Testis cells were incubated in a drop of 50 µg/mL of colchicine in a testis buffer (183 mM KCl, 47 mM NaCl, 10 mM Tris-Cl, 1 mM EDTA, pH 6.8) for 30 min, and then fixed using methanol and acetone [41].

#### Cytochalasin D

Spermatocytes with co-expression of mRFP-actin and GFP-Orbit were dissected from testes and incubated in a testis buffer containing 10 µg/mL of cytochalasin D (Sigma-Aldrich, St. Louis, MO, USA) for 1 h at room temperature before fixation.

### Immunofluorescence

Testis cells were fixed according to the method of Inoue et al. (2004) [41]. For immunostaining, an anti-Orbit antibody was used at a 1∶100 dilution. Two additional primary antibodies, anti-α-spectrin (Developmental Studies Hybridoma Bank, University of Iowa, Iowa City, IA, USA) and anti-phospho-tyrosine (Millipore, Billerica, MA, USA), were used. Microtubules were visualized using immunostaining with anti-α-tubulin (DM1A) (Sigma-Aldrich, St. Louis, MO, USA) and the expression of GFP-β-tubulin [41]. DAPI was used to counterstain DNA in testis cells. Secondary antibodies with a conjugated fluorescent dye (Life Technologies Corp., Carlsbad, CA, USA) were used according to the manufacturer’s instructions. Imaging was performed using an Olympus IX81 fluorescence microscope (Olympus, Inc., Tokyo, Japan), which was additionally fitted with excitation and emission filter wheels (Olympus, Inc., Tokyo, Japan). Fluorescent images were captured using a charge-couple device (CCD) camera (C10600-10B, Hamamatsu Photonics, Shizuoka, Japan). Image acquisition was controlled through MetaMorph version 7.6 (Molecular Devices, Sunnyvale, CA, USA). The images were processed and merged in pseudo color by using MetaMorph. Images of testis cells were also acquired using an Olympus confocal microscope Fv1000 with a 60× objective lens.

### FRET

The fluorescence of CFP and Venus was captured using a cooled CCD camera (Hamamatsu Photonics, Shizuoka, Japan), mounted on an Olympus IX81 microscope with an XF88-2 excitation/emission filter set (OptoScience, Tokyo, Japan). The entire system was controlled using MetaMorph software (Molecular Devices, Sunnyvale, CA, USA). For the quantitation, FRET signals were calculated as the intensity of YFP or Venus (acceptor) fluorescence minus the intensity of CFP (donor) fluorescence by using MetaMorph. FRET/CFP ratio images were created using MetaMorph, and eight colors from blue (lowest) to red (highest) were used to represent the FRET/CFP emission ratio in the intensity-modulated display mode. The minimum and maximum FRET/CFP ratio values were represented in a side bar.

## Results

### Localization of Orbit on Fusomes and Ring Canals of Germline Cysts during *Drosophila* Spermatogenesis

**Figure 1:**
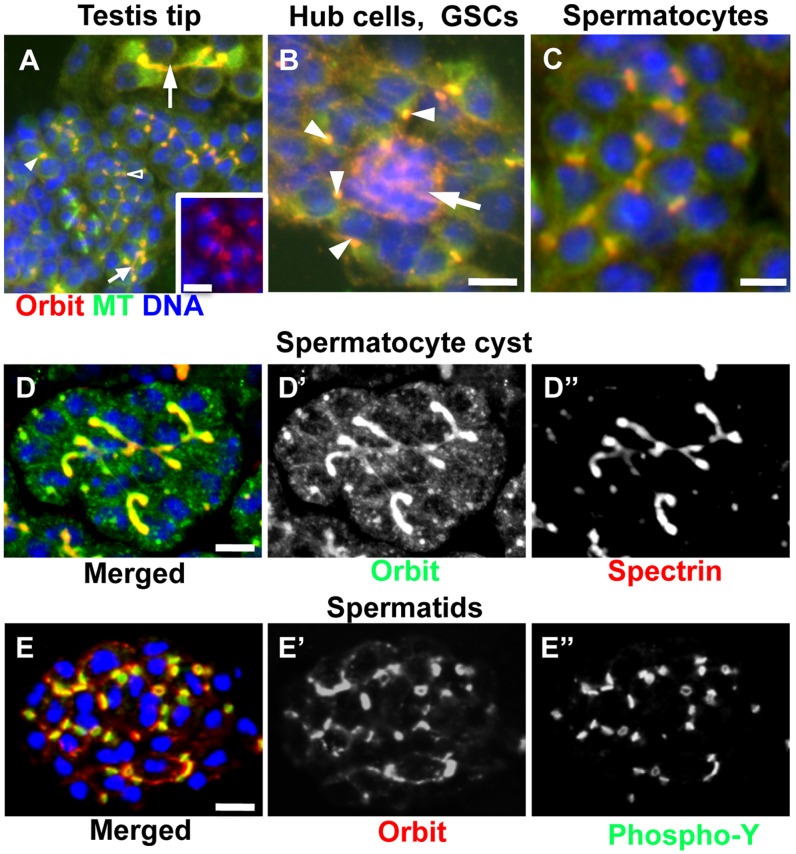
Orbit immunostaining and expression of Orbit fused with fluorescence tags in testis tip cells. (A) Lower magnification view of cells derived from testis tip. Microtubules (green), anti-Orbit immunostaining (red), and DNA (blue) are shown. Orbit is localized on a spectrosome in a 2-cell cyst (smaller filled arrowhead), and on a fusome in an 8-cell cyst (smaller filled arrow). It is also localized on ring canals in a 16-cell cyst (open arrowhead). The larger arrow shows Orbit localization on pieces of fusome formed in a partial spermatocyte cyst at the S2b stage, i.e., the early stage of the growth phase. Inset; a 4-cell spermatogonial cyst at metaphase. (B) Hub cells (arrow) surrounded by several germline stem cells (GSCs). Orbit (red) is highly concentrated in the cytoplasm of hub cells, and on single spectrosomes (arrowheads) in the GSCs. (C) Orbit immunolocalization on ring canals in an early spermatocyte cyst. (D) Expression of GFP-Orbit in an early spermatocyte cyst at the S3 stage, i.e., the middle of the growth phase (green in D, D′). Anti-spectrin immunostaining (red in D, D″) to visualize the fusome is shown. (E) Expression of mRFP-Orbit in a partial spermatid cyst at the onion stage (red in E, E′). Anti-phospho-tyrosine immunostaining (green in E, E″) to visualize ring canals is indicated. Scale bar = 10 µm.

Orbit proteins were previously reported to be localized on germline-specific cellular structures, such as fusomes and ring canals, in *Drosophila* ovarian cysts [13]. The immunolocalization of Orbit during male meiotic divisions was also demonstrated [41]. However, the localization and involvement of Orbit in the premeiotic stage have not previously been characterized in *Drosophila* male cysts. We initially performed immunostaining experiments of testis cells by using an Orbit-specific antibody (Fig. 1A–C). Orbit was first observed in a spectrosome of GSCs, surrounding hub cells (arrowheads in Fig. 1B). Orbit was also concentrated in the cytoplasm of hub cells, which play a role in the generation and transmission of signals for the maintenance of male GSCs (arrow in Fig. 1B). The protein was further detected on mitotic spindles and kinetochores (inset of Fig. 1A). After completion of spermatogonial mitosis, Orbit was concentrated on ring canals (open arrowhead in Fig. 1A). Once a 16-cell cyst of spermatocytes was formed, Orbit was distributed along fusomes until the later stages of the growth phase (larger arrow in Fig. 1A). The fusome in primary spermatocytes persists until the mid-growth stage of spermatocytes, and then gradually regresses and breaks down into pieces [42]. As fusome degradation progresses, Orbit becomes localized around the nuclear envelope [41]. In the present study, during the telophase to early interphase of each spermatogonium, Orbit was distributed on the ring canals (Fig. 1C). Some of the protein temporarily appeared in the axonema of elongated spermatids (Fig. S1). For a more precise and detailed description of cellular localization, we induced the expression of GFP, mRFP, and Venus-tagged Orbit proteins in spermatogonia. We used the UAS/Gal4 system for targeted gene expression in *Drosophila*. To confirm the functionality of these Orbit proteins, we examined whether the induced expression of each protein could rescue a sterile phenotype of hypomorphic *orbit* mutants. The *orbit^7^* homozygotes expressing GFP-, mRFP-, and Venus-Orbit–from spermatogonia to mid-spermatocytes–were fertile males and females, suggesting that the tagged-Orbit acted as a functional protein and exhibited original cellular localization. We induced the expression of fluorescence-tagged Orbit at different developmental stages. The distribution of the fluorescent proteins in a germline cyst coincided with the immunolocalization of Orbit at every developmental stage. In early spermatocyte cysts, after completion of the fourth round of spermatogonial divisions, the GFP-Orbit remained localized on ring canals connecting spermatocytes within a cyst (Fig. 1C). As spermatocytes initiated their growth phase, the fusomes elongated through the ring canals, towards all spermatocytes in a cyst. Orbit shifted its localization from ring canals to fusomes during the early growth phase. As each spermatocyte increased its cell volume during the growth phase, the fusomes extended and increased their length and width. Orbit was uniformly distributed along the fusomes during the growth phase (Fig. 1D). Prior to meiotic initiation, the extended and branched structures of the fusomes disintegrated into smaller fragments, and Orbit was localized on the remaining fusome pieces (arrowhead in Fig. S2A). During meiotic divisions, Orbit was localized around the nuclear membranes during the prophase (Fig. S2A) and associated with spindle envelopes and kinetochores from the prometaphase to the metaphase (Fig. S2B). At the anaphase, Orbit was concentrated on interior central spindles (Fig. S2C). By the telophase, Orbit had accumulated on the prospective cleavage furrow region and contractile rings; the protein remained on the contractile rings during cytokinesis (Fig. S2D). We observed the same localization of Orbit by immunostaining of meiotic cells using the Orbit antibody [41]. After completion of male meiosis II, fusome structures containing Hts proteins regenerated into onion-stage spermatids [7]; however, we did not detect the distinct localization of Orbit on post-meiotic fusome structures (Fig. 1E).

### Close Association of Orbit with F-actin in Fusomes

**Figure 2:**
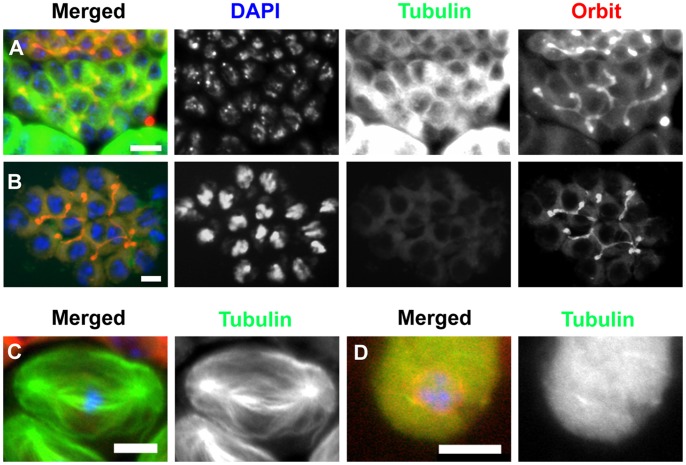
Inhibition of microtubule polymerization does not influence the maintenance of Orbit localization on fusomes. Green is GFP-tubulin, red is mRFP-Orbit, and blue is DAPI staining. (A) An early spermatocyte cyst incubated without colchicine. Note the cytoplasmic microtubule structures and distinct Orbit localization on growing fusomes. (B) An early spermatocyte cyst treated with colchicine; a branched fusome structure is intact. (C, D) Microtubule degradation by colchicine treatment: (C), a metaphase I cell without colchicine treatment; (D), a meiotic cell treated with colchicine. Scale bar = 10 µm.

Orbit has been identified as a microtubule-associated protein [35]. Therefore, we examined whether Orbit localization on fusomes is dependent on microtubules. Testis cells were incubated for 30 min in a buffer containing 50 µg/ml of colchicine (Fig. 2A, B). We confirmed the complete degradation of spindle microtubules and astral microtubules after colchicine treatment (Fig. 2C, D). Next, we examined whether Orbit localization on fusomes changed in the same testis preparation. We observed that fusome localization of Orbit was maintained after colchicine treatment (Fig. 2B). Therefore, we concluded that Orbit localization on the fusome is independent of microtubules and that microtubules are not essential for the maintenance of the fusome structure.

**Figure 3:**
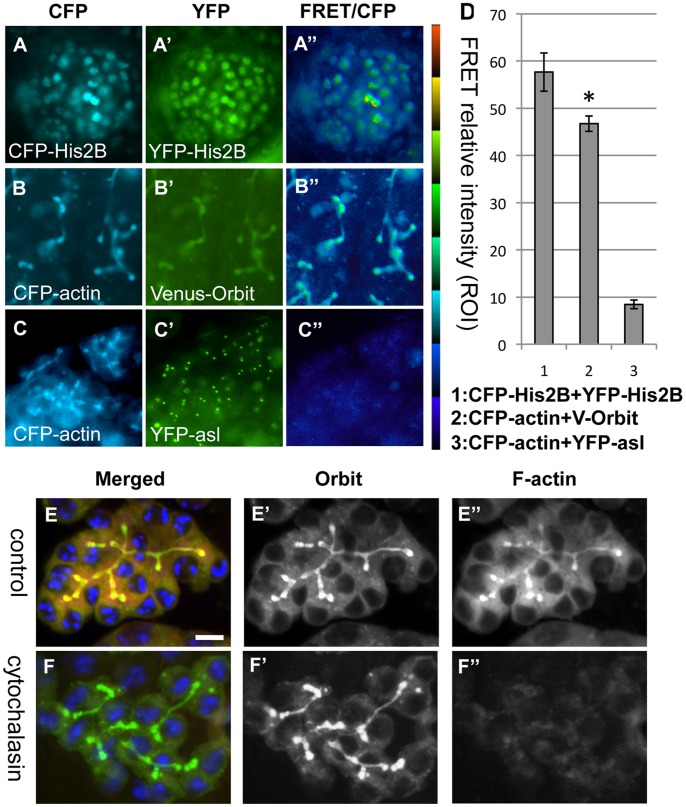
Close association of Orbit with F-actin contained in a fusome of a spermatocyte cyst. (A–C″) CFP and YFP fluorescence images, and fluorescence resonance energy transfer (FRET)/CFP emission ratio images in spermatocytes. (A–A″) Positive control for FRET experiment in early spermatocytes. Co-expression of CFP-histone 2B (A) to YFP-histone 2B (A′) was induced in early spermatocytes using the UAS/Gal4 system. (A″) A FRET/CFP emission ratio was calculated using MetaMorph software. The distribution of the ratio between the minimum value (darkest blue) and maximum value (lightest red) is represented by the 8-step color indicator in the intensity-modulated display mode. Note the distinct YFP emission in nuclei of early spermatocytes after CFP excitation. It is well known that histone H2B forms a dimer in a nucleosome, and therefore, the YFP emission is generated because of FRET from CFP to YFP. (B–B″) CFP and FRET/CFP ratio images of spermatocytes with co-expression of Venus-Orbit and CFP-actin. CFP (B) and FRET/CFP ratio images (B″) of spermatocytes with co-expression of Venus-Orbit and CFP-actin. (C–C″) A spermatocyte with co-expression of CFP-actin (C) and YFP-Asl (C′), as a negative control for the FRET experiment. (D) The average FRET relative intensity in nuclei of spermatocytes with co-expression of CFP-Histone 2B and YFP-Histone 2B, fusomes of spermatocytes with co-expression of CFP-actin and Venus-Orbit, and fusomes in spermatocytes with co-expression of CFP-actin and YFP-Asl. The FRET relative intensity was calculated and represented according to [44]. (E) F-actin and Orbit are components of fusomes extending in a spermatocyte cyst. RFP-actin (red), GFP-Orbit (green), and DNA (blue) are shown. (F) F-actin depolymerization induced by treatment with cytochalasin D does not influence Orbit localization on fusomes, or maintenance of the fusome structure. Scale bar = 10 µm.

Male fusomes and early ring canals in spermatogonia and spermatocytes contain F-actin (Fig. 3E). Therefore, we examined whether Orbit was localized on fusomes through binding with F-actin. We initially induced co-expression of CFP-Histone 2B and YFP-Histone 2B in early spermatocytes (Fig. 3A–A″). When the spermatocytes were illuminated via the excitation of CFP, we measured the generated fluorescence by using FRET from CFP to YFP. This fluorescence is considered to be a consequence of dimer formation between CFP-Histone 2B and YFP-Histone in a nucleosome. We subsequently induced co-expression of CFP-actin and Venus-Orbit in premeiotic spermatocytes (Fig. 3B–B″). When the spermatocytes were illuminated via the excitation of CFP, we measured the generated fluorescence from CFP to Venus by using FRET. We detected a strong FRET signal on fusomes in premeiotic spermatocyte cysts (Fig. 3B″). As a negative control, we confirmed the absence of a FRET signal in the cytoplasm of spermatocytes with co-expression of CFP-actin and YFP-Asl, which is a centrosome protein (Fig. 3C–C″). Our results confirmed that CFP-actin and Venus-Orbit are co-localized within a close distance (10 nm), where effective FRET can occur. It is likely that these two proteins are closely associated with each other on the fusome.

### Lack of Effect of F-actin Depolymerization on Fusome Structure and Orbit Localization on the Fusome

We examined the effect of F-actin depolymerization drugs on the fusome localization of Orbit. Cytochalasin D is an inhibitor of actin polymerization and causes F-actin depolymerization. After treatment with cytochalasin D, the F-actin structure contained in the fusome disintegrated (Fig. 3F″). However, the entire fusome structure was maintained (data not shown) and, surprisingly, Orbit remained localized on the fusomes (Fig. 3F′). Furthermore, inhibition of actin polymerization influenced neither fusome organization nor Orbit localization on the germline-specific cytoskeleton. We observed the same pattern of results after treatment with latrunculin A, another F-actin depolymerizing drug (Fig. S3D). Therefore, we propose that Orbit localization on the fusome does not require stable F-actin, which is abundantly contained in the cytoskeleton.

### Dependence of Fusome Localization and Ring Canal Localization on the Different Functional Domains of Orbit

**Figure 4:**
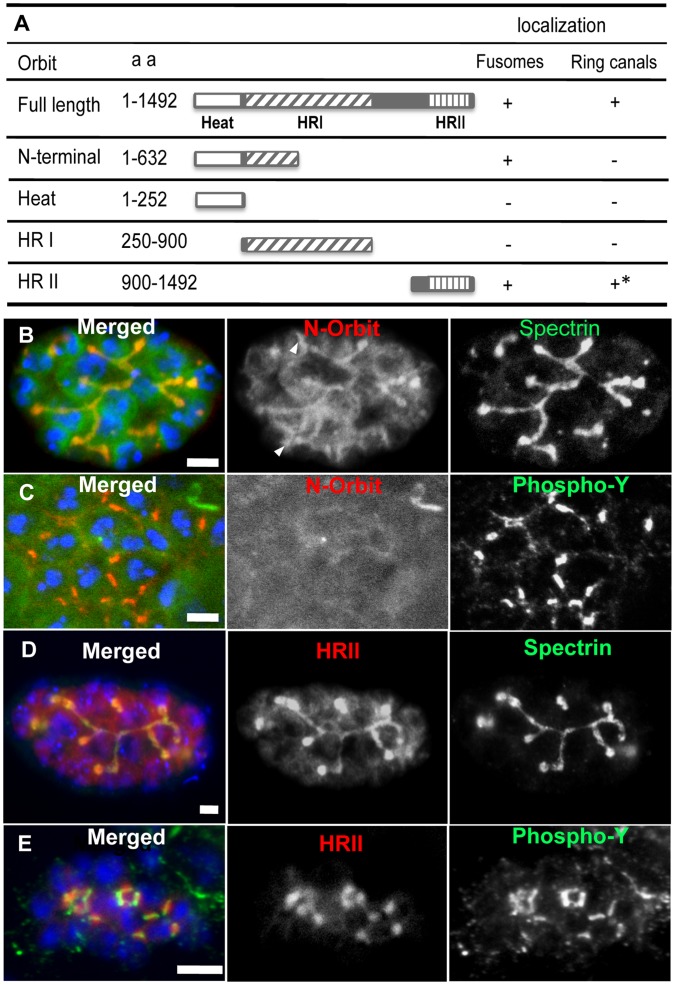
Conserved regions of Orbit are required for localization on fusomes and ring canals. (A) Schematic representation of the conserved Orbit regions and localization of each domain on fusomes or ring canals. *HRII is localized inside ring canals, but is not restricted on the ring canal rims as shown for the wild-type Orbit. (B) The N-terminal region alone fused with GFP tag (green) is localized on fusomes stained with anti-spectrin antibody (red). Single-channel representation of N-Orbit fluorescence (middle). Single-channel representation of anti-spectrin immunostaining (right). The truncated protein can be additionally localized around each nucleus (arrowheads). (C) The N-terminal region fused with GFP tag (green) is not localized on ring canals recognized by anti-phospho-tyrosine antibody (red). Single-channel representation of N-Orbit fluorescence (middle) in an early-stage spermatocyte cyst. Single-channel representation of anti-phospho-tyrosine immunostaining (right). (D) In an early spermatocyte cyst at the S2a stage, the HRII region (red) is localized on fusomes (green). Single-channel representation of HRII-Orbit fluorescence (middle). Single-channel representation of anti-spectrin immunostaining (right). (E) The HRII region (red) is localized on fusome plugs formed in midbody between separated sister nuclei, but is not perfectly overlapped with ring canal rims stained with anti-phospho-tyrosine (green) in early spermatocyte cysts. Single-channel representation of RFP-HRII fluorescence (middle). Single-channel representation of anti-phospho-tyrosine immunostaining (right). DNA staining (blue). Scale bar = 10 µm.

To elucidate the regulatory mechanism of Orbit localization during spermatogenesis, we examined the regions required for its localization on fusomes and ring canals. In previous studies, three regions were conserved among Orbit/CLASP family proteins: Heat (1–252 aa), HRI (250–900 aa), and HRII (900–1492 aa) (Fig. 4A) [35], [36]. We induced the expression of these Orbit regions fused with GFP or mRFP in testis cells and observed their localization in spermatocyte cysts (Fig. 4B-E). The Heat domain has previously been found among Huntington, Elongation Factor 3, PR65/A, and TOR proteins; however, its biochemical role has not been elucidated [43]. In the present study, the Heat domain did not show distinct localization in early- to late-stage premeiotic spermatocytes. The HRI region did not co-localize with microtubules in meiotic cells, but was identified as a domain sufficient for microtubule association and bundling in cultured cells (Fig. S5B). In addition, the HRI region did not localize on fusomes or ring canals. Unlike the HRI region, the N-terminal region (1–632) contained amino acid sequences sufficient for microtubule binding *in vitro* [35] and in meiotic cells. The N-terminal region was localized on growing fusomes in early spermatocytes and extended fusomes in later spermatocytes (Fig. 4B); however, specific localization on ring canals was not observed (Fig. 4C). The HRII region, which corresponded to the C-terminal one-third of Orbit, showed localization on fusomes (Fig. 4D). In addition, the HRII polypeptides were accumulated around ring canals, but were not perfectly overlapped with ring canal rims visualized using anti-phospho-tyrosine immunostaining (Fig. 4E). Instead, they appeared to be localized on cellular structures known as fusome plugs, which are newly formed on the midbody at the telophase and develop into regularly branched structures in early-spermatocyte cysts (Fig. 4E). The HR II polypeptides were not found on matured ring canals. Our results suggest that Orbit possesses two separate domains, each of which can localize on fusomes in spermatocytes. One such domain corresponds to the N-terminal region with microtubule-binding activity *in vitro* [35]. The polypeptides appeared to distribute along central spindle microtubules at anaphase I. A considerable amount of these polypeptides remained in the cytoplasm, suggesting a relatively low affinity for fusomes. In addition, the N-terminal region polypeptides did not show distinct localization on ring canals in spermatogonia and spermatocytes (Fig. 4A). The accumulation of the N-terminal fragment of Orbit on the fusome is much weaker and more diffuse than that of HRII (Fig. 4B). Another fusome-binding domain was mapped on the HRII region and was not localized on microtubule structures in spermatogonia, spermatocytes, or cultured cells (data not shown). Taken together, our results indicate that Orbit regions essential for localization on microtubules and fusomes do not overlap each other; this finding is consistent with fusome localization being independent of microtubules.

### Effect of an *Orbit* Mutation on the Proper Formation of Fusomes and Ring Canals

**Figure 5:**
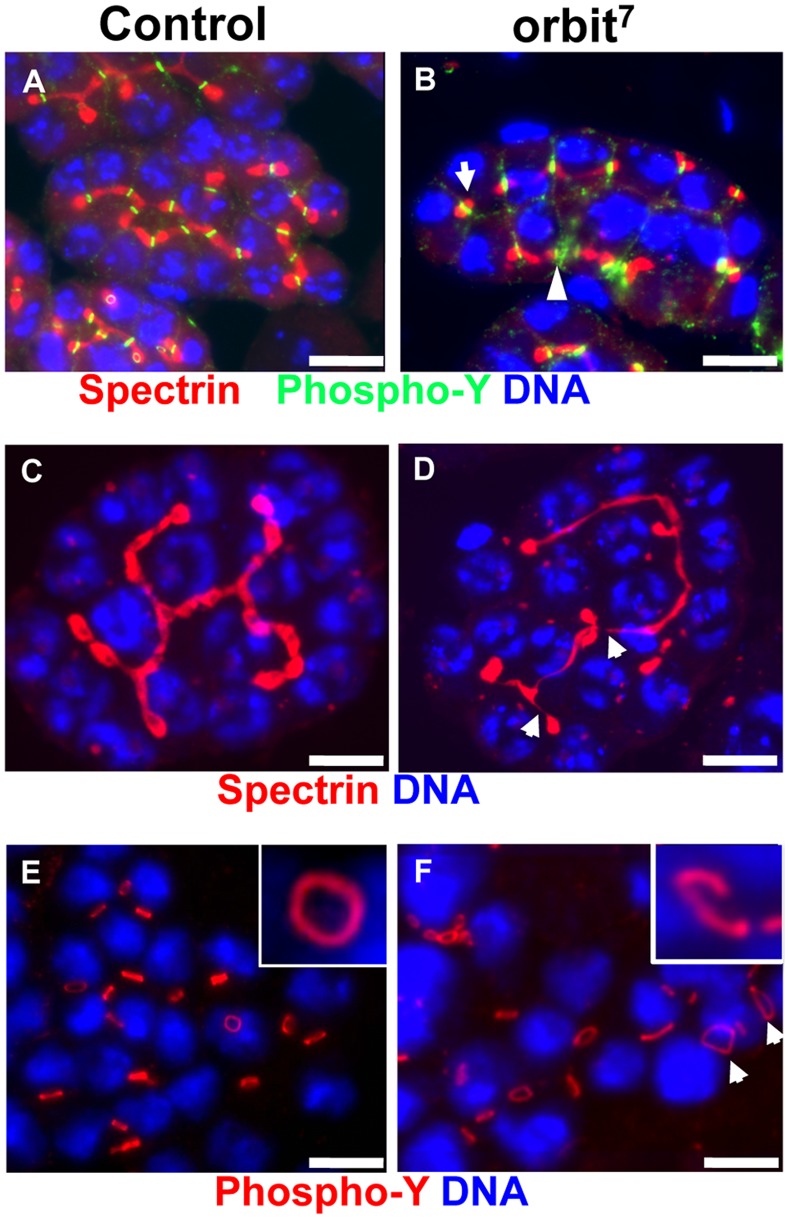
The *orbit* gene is required for development of fusomes and ring canals in spermatocyte cysts. (A, B) Immunostaining of early spermatocyte cysts from normal males and from *orbit^7^* mutant males by using anti-α-spectrin antibody for fusome visualization (red) and anti-phospho-tyrosine for ring canal observation (green). DNA staining (blue). Note the abnormal fusomes, which failed to elongate (arrow) or branch in the mutant cysts. The ring canal marker failed to be incorporated in the lumen of ring canals (arrowhead). (C) A normal branched fusome structure with constant thickness in a wild-type early spermatocyte cyst at the S2 stage. (D) A less-branched fusome structure in the mutant spermatocyte cyst. The abnormal fusome becomes thinner in places or disconnected (arrows). (E) Immunodetection of ring canals in early spermatocyte cysts using anti- phospho-tyrosine antibody. Note the formation of ring canals with constant diameter between every nucleus (blue) in wild-type spermatocytes. Note also that normal ring canals are shaped by a continuous hollow structure (inset). (F) In early spermatocytes from *orbit^7^* mutant males, disconnected ring canals are observed in the mutant cysts (inset). Abnormal ring canals with larger diameters (arrows) are observed in early spermatocytes from *orbit^7^* mutant males. Scale bar = 10 µm.

Previous studies have reported that fusome growth was compromised in ovarian egg chambers from hypomorphic *orbit* mutants [13]. In the present study, Orbit was localized on elongating fusomes in spermatogonia and on enlarged fusomes in early spermatocytes (Fig. 1). Therefore, we examined the requirement of Orbit for fusome development in premeiotic spermatocyte cysts. We observed that, in wild-type cysts, fusomes extended into every cell within a cyst, through ring canals at the early spermatocyte stage (Fig. 5A, C). The newly formed fusome plugs connected with pre-existing fusomes and developed into regularly branched structures in early spermatocyte cysts (Fig. 5A, C). By contrast, in hypomorphic *orbit^7^* mutant cysts, spermatocyte cysts containing abnormal fusomes were observed (eight abnormal cysts out of 70 spermatocyte cysts examined). The fusome plugs were formed inside ring canals, but were not continuously connected with existing fusomes (arrow in Fig. 5B). In mutant cysts, thinner fusome structures linked to fusome plugs were often observed (Fig. 5D). Spermatocytes within a mutant cyst were aligned (see front and second cell rows in the 16-spermatocyte cyst shown in Fig. 5B). In early mutant spermatocyte cysts, mutant fusome pieces appeared to be distributed as a broken line (Fig. 5B) or were uninterrupted but less branched (Fig. 5D). These abnormal fusomes had a similar appearance to the abnormal fusome structures previously observed in the egg chambers of *orbit^7^* females [13]. Furthermore, Orbit was found on ring canals in a cyst, from the spermatogonium to the spermatocyte stage (open arrowhead in Fig. 1A, Fig. 1C). Ring canals of various diameters were frequently observed in the mutant cysts (arrows in Fig. 5F), whereas ring canals of constant diameter were formed in wild-type cysts (Fig. 5E). Abnormal ring canals were observed in three out of 15 early spermatocytes cysts from mutant males, but no cysts out of 30 wild-type cysts. The presence of larger ring canals in the mutant cysts suggests that spermatogonial cytokinesis was arrested midway (arrows in Fig. 5F). This is consistent with the cytokinesis phenotype in meiotic cells from the *orbit* mutant [41]. In addition, interrupted ring canals were observed using immunostaining with phospho-tyrosine antibody (inset of Fig. 5F) or anillin antibody (arrowhead, Fig. S4).

### Orbit Accumulation on One of Two Spindle Poles and on Fusomes around the Pole during Spermatogonial Mitosis

**Figure 6:**
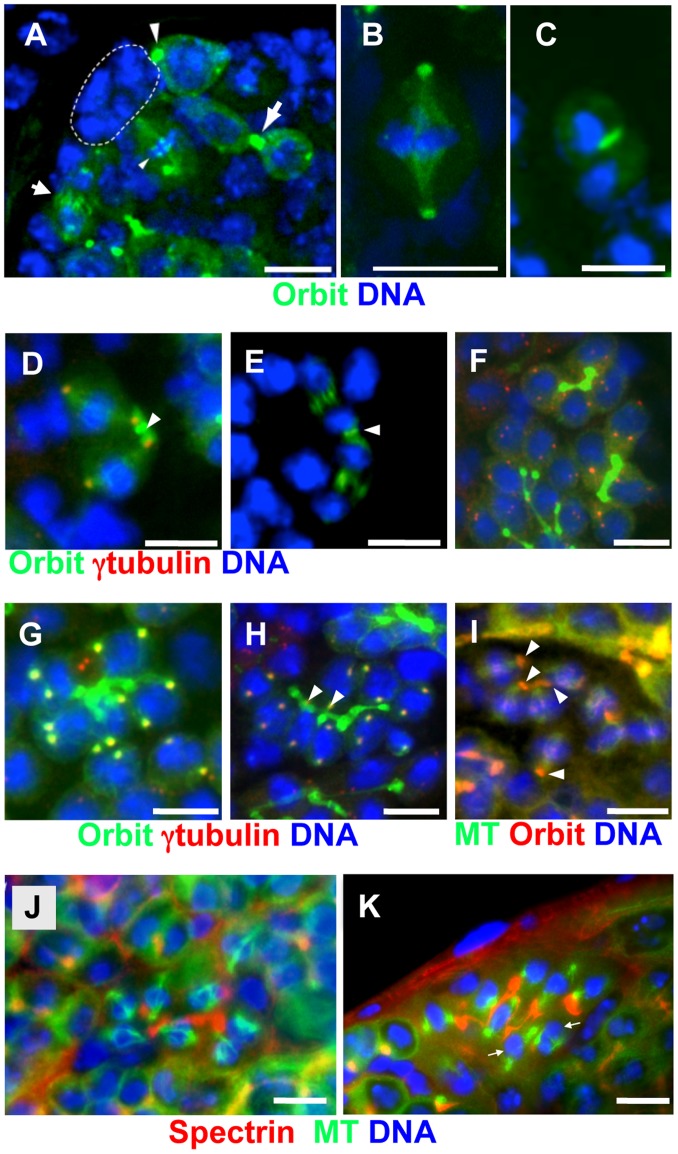
Dynamic alteration of Orbit localization in germline stem cells (GSCs) and spermatogonial division. (A–H) GFP-Orbit (green) and DNA (blue) are shown. (D, F, G, H) anti-γ-tubulin immunostaining (red). (I) GFP-tubulin (green), anti-Orbit immunostaining (red), and DNA (blue). (J, K) GFP-tubulin (green), anti-spectrin antibody immunostaining (red), and DNA (blue). (A) Tip of a testis from a *nos-Gal4>UAS-GFP-Orbit* male. Hub cells are encircled by a dotted line. Orbit is localized on the spectrosome of a GSC (larger arrowhead), kinetochores of a GSC at metaphase (smaller arrowhead), central spindle microtubules of a presumptive GSC (smaller arrow), and fusome plug formed at the midbody of a presumptive GSC (larger arrow). (B) A gonialblast undergoing the first division at metaphase. (C) Late anaphase of a gonialblast. (D) In the second spermatogonial division, two centrosomes (red) are connected by a single spectrosome (arrowhead). Orbit is more abundant on spectrosomes and around centrosomes attached to the spectrosome, than on distal centrosomes. (E) A 2-cell cyst of dividing spermatogonia at anaphase. Note the contractile ring (arrowhead) between two cells. (F) Two 4-cell cysts and part of a 16-cell cyst at interphase. Most of the centrosomes (red) are not associated with fusomes at interphase. (G) An 8-cell cyst in which γ-tubulin foci (yellow) have become evident at the onset of the third mitotic division. (H) Prophase to prometaphase of the third spermatogonial division. Every centrosome pair (red) orients towards the fusome, and one of the two centrosomes (arrowheads) is captured by the fusome. (I) Immunostaining of an 8-cell cyst at metaphase. Orbit is localized on kinetochores and part of the fusome (arrowheads). (J, K) An 8-cell spermatogonial cyst undergoing mitosis; (J) normal control male and (K) *orbit^7^* mutant male. The mutant fusome appears to be disconnected. At least two mitotic spindles (arrows) have been detached from the fusome.

Previous studies have reported the immunolocalization of Orbit in male meiotic cells [41]. However, Orbit localization in male GSCs and spermatogonia at the mitotic stages is difficult to observe because of the considerably low frequency of dividing cells. In the present study, we used the expression of GFP-tagged Orbit, rather than immunostaining, to observe Orbit throughout the cell cycle. Orbit localization first became visible on the spectrosome at the interphase of GSCs (larger arrowhead in Fig. 6A). In female GSCs, the spectrosome is associated with the apical spindle pole at the interphase [19]. By contrast, male GSCs contain spectrosomes that are associated with the distal spindle pole [34]. In accordance with previous studies, we observed that Orbit was also localized on cell division apparatuses, such as spindle microtubules, kinetochores, and centrosomes, in other cell types and at different developmental stages (smaller arrowhead and smaller arrow in Fig. 6A) [35], [41]. At the telophase, Orbit accumulated on the newly formed fusome plugs described above (larger arrow in Fig. 6A).

In the first division of the gonialblast, which corresponds to a cell that lies at the tip of the testis region but does not contact with hub cells, Orbit was distributed along spindle microtubules and accumulated on both spindle poles at the metaphase (Fig. 6B). After completion of the first spermatogonial division, Orbit was found on contractile rings and thereafter remained localized on the ring canals (Fig. 6C), as previously demonstrated in male meiotic division [41]. Spectrosome localization of the protein was not detected during gonialblast division. Subsequently, the protein reappeared on a spectrosome formed in a ring canal between two spermatogonia in a 2-cell cyst (Fig. 6D). In the 2-cell cyst, one of two centrosomes appeared to be captured by a growing fusome, which extended through the first ring canal; Orbit was concentrated on the growing fusome in a ring canal (arrowheads in Fig. 6D, E).

Next, we examined whether one of the duplicated centrosomes was retained on the growing fusome, because only mother centrosomes remained attached to the hub-side cortex in male GSCs. In spermatogonial cysts at the interphase, most centrosomes were distributed randomly, i.e., independently on fusomes (Fig. 6F). Orbit was not associated with centrosomes before this stage. When γ-tubulin foci were marked, the timing appeared to correspond to the onset of gonial division, and Orbit accumulated on both centrosomes in each cell (Fig. 6G). From the prophase to the prometaphase, each pair of centrosomes appeared to orient towards the fusome (Fig. 6H). In some cells, one of the two centrosomes was attached to the fusome (arrowheads, Fig. 6H). In a metaphase cyst, Orbit was locally distributed around a small region close to one of the two centrosomes, which may correspond to part of the fusome (Fig. 6I). This Orbit distribution in mitotic 8-cell cysts was consistent with the distribution observed in a 2-cell cyst (Fig. 6D). At this stage, Orbit was not found along the fusome (Fig. 6I), although anti-α-spectrin immunostaining indicated the presence of this germline-specific cytoskeleton (Fig. 6J).

Unlike in meiotic divisions, fusomes were not degraded prior to every spermatogonial division. At the mitotic stage of spermatogonia, the interphase localization of Orbit was changed from fusomes towards spindle microtubules and around centrosomes. To elucidate the cellular role of Orbit during cell division of spermatogonia and spermatocytes, we examined whether the *orbit* mutation could influence synchronized mitosis in a spermatogonial cyst. We observed that, in a wild-type 8-cell cyst, one of two spindle poles appeared to attach onto the fusome at the metaphase (Fig. 6J). Conversely, in the *orbit^7^* mutant, some mitotic figures (arrows in Fig. 6K) lost their connection with fusomes, which became narrower in the middle. In at least two cells within the cyst, the mutant fusomes failed to capture either of the two spindle poles. We reproducibly observed the phenotype in three out of 11 mitotic spermatogonial cysts from the hypomorphic mutant males. Therefore, it is likely that Orbit plays an important role in organizing the mitosis of spermatogonia, through capturing one of the spindle poles onto the fusome.

## Discussion

To identify an essential factor that regulates synchronous mitosis of *Drosophila* spermatogonia and the formation of 16-cell cysts, we examined the cellular localization and role of a microtubule-associated protein, Orbit, in male germline cyst formation. We observed that Orbit was localized on cell division apparatuses during spermatogonial mitosis and on the germline-specific cytoskeleton, which is required for cyst formation, at the interphase. In GSCs, distinct Orbit localization was first observed on spectrosomes. After cytokinesis, the protein remained localized on ring canals. Thereafter, it was found on spectrosomes and fusomes in spermatogonia and spermatocytes at the interphase. Orbit was initially identified as a microtubule-binding protein; however, its fusome localization was not altered by microtubule depolymerization. Instead, our FRET experiments suggested that Orbit is closely associated with F-actin, which is abundantly contained in fusomes of premeiotic spermatocytes. Surprisingly F-actin depolymerization influenced neither fusome localization nor Orbit localization on the germline-specific cytoskeleton. By mapping essential domains to determine Orbit localization, we revealed that at least two non-overlapping regions were sufficient for fusome localization in spermatocytes. Genetic analyses of hypomorphic *orbit* mutants showed that Orbit was important for cyst formation in spermatogenesis. Mutant spermatocytes possessed disintegrated fusomes, which failed to elongate or branch, and abnormal ring canals. Orbit was concentrated around centrosomes at the initiation of mitosis, and its reduction disrupted the connection between spindle poles and fusomes. Taken together, our findings indicate that Orbit plays an important role in organizing spermatogonial mitosis, through the interaction of fusomes and spindle poles, and in fusome formation at the interphase.

### Possible Role of Orbit as an Actin-associated Protein on Fusomes at the Interphase in *Drosophila* Spermatocytes

Orbit is recognized as a microtubule-binding protein [35], [36]. Accordingly, we examined whether Orbit becomes localized on fusomes through its microtubule-binding activity. In contrast to previous studies on female fusomes [31], we observed that male fusomes were not closely associated with microtubules at the interphase in either spermatogonia or premeiotic spermatocytes. In addition, we demonstrated that colchicine treatment, which resulted in microtubule depolymerization, did not influence Orbit localization on fusomes. Thus, it is unlikely that Orbit becomes localized on fusomes by means of its microtubule-binding activity. In accordance with this conclusion, we revealed that the HRII region, which corresponds to the C-terminal one-third of Orbit, showed localization on fusomes. Instead, our FRET experiments indicated that Orbit proteins are localized on fusomes via close association with F-actin, rather than with microtubules. Orbit proteins are also known to be associated with a contractile ring consisting of F-actin [41]. We previously reported that Orbit is localized on fusomes and ring canals, which connect cystocytes in the egg chamber [13]. This is consistent with the mouse ortholog CLASP2, which possesses a binding domain for F-actin in the cell cortex [44]. Therefore, it is possible that Orbit plays an additional role as an actin-associated protein *in vivo*.

In the present study, we demonstrated that the N-terminal region and C-terminal HRII domain could each be localized on fusomes. In a separate study, we also showed that the HRII domain could be localized on contractile rings (Kitazawa et al., submitted). These two regions do not overlap, and therefore, Orbit must possess at least two separate domains that are sufficient for actin association. The actin-binding activity of CLASP2 has been mapped to the Dis1/TOG domain at its N-terminus [44]. However, the corresponding HEAT domain of *Drosophila* Orbit (1–252) was insufficient for fusome localization. The N-terminal region (1–632) can be localized on fusomes, but to a lesser extent than the entire Orbit protein. Thus, the HEAT domain of Orbit must be lacking essential amino acid sequences for fusome localization. Mouse CLASP2 has an additional domain sufficient for actin binding in the middle of the HRI-corresponding region; however, no actin-binding activity is present on the corresponding domain of *Drosophila* Orbit. Thus, it is likely that the alignment of actin-binding domains differs between Orbit and CLASP2 orthologs.

Surprisingly, treatment with cytochalasin D or latrunculin A, which induce depolymerization of F-actin, did not influence fusome organization. By contrast, *orbit* mutations significantly affected fusome elongation, indicating that Orbit may play an important role in maintaining the fusome structure. We speculate that Orbit may bind to an essential component in the fusome, other than microtubules or F-actin. Several proteins have been reported to be associated with male fusomes, e.g., actin-binding proteins, α-spectrin [29], [42], Hts [29], microtubule motor protein, KLP61F [29], and Par-1 [26]. Some regulatory proteins, such as histone methyltransferase G9a [45] and cyclin A [34], are also known to interact with fusomes. None of these proteins has been characterized as a scaffold component in fusomes; however, α-spectrin has been shown to provide support to the cytoskeleton network of the cell cortex [46], [47]. It would be interesting to investigate whether a similar spectrin cytoskeleton network functions as a scaffold structure for male fusomes, independently of F-actin.

We also demonstrated that Orbit became localized on contractile rings, which constitute another F-actin-enriched structure, and ring canals in spermatocytes. The N-terminal domain, where the fusome-binding domain is mapped, was insufficient for ring canal localization. The N-terminal domain showed less effective localization on fusomes, indicating that it is insufficient for full actin-binding activity. Alternatively, separate domains that interact with microtubules might be required for ring canal localization.

### Importance of Orbit in Ring Canal Formation and Fusome Construction

In the present study, we observed larger ring canals in spermatogonial cysts from *orbit* mutants. Our finding suggests the cessation of cleavage furrow ingression by midway of mitotic cytokinesis and is consistent with previous reports of the failure of cytokinesis in mutant male meiotic cells [41]. After completion of cytokinesis in spermatogonial mitosis, the contractile rings stabilize and transform into ring canals [7], [48], [49]. Subsequently, proteins containing phospho-tyrosine appear on the contractile rings. By immunostaining with phospho-tyrosine antibody and anillin antibody, we visualized disconnected ring canals in the mutant cysts, suggesting that some contractile ring components were not distributed properly on the cleavage furrow region. Some contractile ring components and new membrane vesicles have previously been shown to be transported into the cleavage furrow region in a microtubule-dependent manner [50]. The transport of these components towards the cleavage furrow may be inhibited in *orbit* mutants. Alternatively, similar to larger ring canals, abnormal ring canal structures may be a secondary defect of abnormal spermatogonial mitosis.

In *orbit* mutant females, inner-rim ring canal proteins, e.g., F-actin, filamin, and HtsRC, were previously shown to obstruct the ring canals [13]. In the present study, we did not observe similar obstruction in *orbit* mutant males. The inner-rim markers of female ring canals are not contained in the male ring canal [7]. Another female ring canal marker Kelch, which was missing from the mutant ring canals, is not contained in wild-type males. Inner-rim markers were not available for male ring canals, and therefore, we did not examine the inner lumen of male ring canals.

In less-defective spermatogonial cysts from the *orbit* mutants (Fig. 5D), we observed a continuous fusome, which appeared to turn less frequently. At the end of each cystocyte division in the egg chambers, nascent ring canals have been shown to move towards the center of the cyst [17]. This ring canal migration is considered as important for the folding of cystocytes and the generation of a compact germline cyst. The migration also facilitates fusion of newly developed fusome plugs (larger arrow in Fig. 6A) with pre-existing fusomes. Eventually, a continuous and regularly turned fusome structure is constructed. The continuous, but less turned, fusomes in the *orbit* mutant suggest that fusome extension was present but that ring canal migration failed to occur properly. The regulatory mechanism for ring canal migration has not been discovered; however, it is possible that Orbit is involved in ring canal migration. A similar phenotype has been reported in loss-of-function mutants for the microtubule plus-ended motor KLP61F [29]. Orbit is known to be a plus-ended enriched protein, and therefore, it would be interesting to examine the genetic interaction between KLP61F and Orbit in male fusome formation.

### Possible Role of Orbit in Synchronous Spermatogonial Mitosis to Construct a Germline Cyst

The anchoring of one of the two centrosomes in germline cells onto the fusome allows spindle microtubules to establish a fixed orientation towards the fusome. In female GSCs, the spectrosome, which is a precursor of the fusome, lies at the cortex adjoining the cap cells. One of the duplicated centrosomes makes contact with the spectrosome and directs spindles towards the cap cells [20], [51]. Meanwhile, male GSCs have a distinct mechanism for orienting the mitotic spindle [52]. The mother centrosome continues to be captured at the hub-side cortex, while the daughter centrosome is free to migrate towards the opposite side to generate a bipolar spindle [53]. In the present study, we demonstrated that one of the centrosomes appeared to be closely associated with elongating fusomes during spermatogonial mitosis. A similar interaction between spindle poles and growing fusomes was previously observed in female cystocyte division [18], [20]. In other studies of female GSCs, duplicated centrosomes were found to be initially positioned at random [34], [52]. Meanwhile, in mitotic ovarian cysts, one of two centrosomes was shown to be closely associated with the fusome [13]; however, the mechanism of capture of one centrosome on the fusome remains unclear. In the present study, both of the duplicated centrosomes were initially not associated with fusomes (Fig. 6). After bipolar organization of the centrosomes was established, one of the two centrosomes appeared to be oriented on the fusome until the prometaphase. We demonstrated that Orbit is localized on the proximal spindle pole and revealed a connection between the fusome and centrosomes in some mitotic spermatogonial cysts from *orbit* mutants. Therefore, we propose that Orbit may be involved in anchoring centrosomes to the fusome. Microtubules have not been found on or around fusomes in spermatogonial and primary spermatocyte cysts. Interestingly, we observed fibrous structures (as visualized by GFP-Orbit) that link the centrosomes and fusomes at the prophase. During spermatogonial mitosis, Orbit accumulated on one of spindle poles and on the fusome around the pole. Therefore, it would be worthwhile investigating the possible role of Orbit in anchoring single spindle poles.

## Supporting Information

Figure S1 Immunostaining with anti-Orbit antibody appears temporary before basal body foci become prominent in elongated spermatids. (A–C) Immunostaining with anti-Orbit antibody (red), GFP-tubulin (green), and DNA staining (blue). (A) A cyst of elongated spermatids without distinct basal body foci. (A′) A merged image without red-channel presentation. The Orbit antibody recognizes a junction between the nucleus and axonema, in which basal bodies should be present. In a later elongated spermatid stage, the GFP-tubulin foci, corresponding to basal bodies, become conspicuous. The foci are not yet prominent in these spermatids. (B) An upper spermatid cyst in which spermatid individualization has been partially initiated is not stained with Orbit antibody. Immunostaining can be seen in the lower cyst, which contains earlier spermatids without basal body foci. (B′) A merged image without red-channel presentation. (C) In another two cysts of elongated spermatids, in which each spermatid appears to be tightly assembled, basal body foci are not stained with Orbit antibody. Scale bar = 10 µm.
(TIF)

Figure S2 Cellular localization of Orbit with fluorescence tags in male meiotic cells. (A, C, D) Primary spermatocytes with expression of GFP-Orbit (green) at prophase (A), late anaphase (C), and cytokinesis stage (D). (B) Primary spermatocytes with mRFP-Orbit (red) at metaphase. Immunostaining with anti-tubulin antibody (red for A, C, D; green for B) was carried out to determine meiotic stages. Arrowhead in A indicates the remnants of the fusome, after its degradation before the initiation of meiosis. Scale bar = 10 µm.
(TIF)

Figure S3 Inhibition of actin polymerization does not influence the maintenance of Orbit localization on fusomes. (A, D) RFP-actin (red), GFP-Orbit (green), and DNA (blue). (A–C) A control spermatocyte cyst in which F-actin and Orbit are components of extending fusomes. (D–F) F-actin depolymerization induced by treatment with latrunculin A influences neither Orbit localization on fusomes nor maintenance of the fusome structure. Scale bar = 10 µm.
(TIF)

Figure S4 Abnormal organization and distribution of ring canals, visualized by immunostaining of anillin (a ring canal component), in a spermatocyte cyst from *orbit^7^* mutant males. (A) In wild-type spermatocytes, normal (control) ring canals with constant diameter are distributed between every nucleus (blue). Anillin (red) is localized in nuclei and matured ring canals. (B) In spermatocyte cysts from *orbit^7^* mutant males, ring canals with various diameters are observed. Two of the ring canals are distributed close to each other. Scale bar = 10 µm.
(TIF)

Figure S5 The full-length Orbit protein and HRI region are localized on microtubule bundles, induced by overexpression of the polypeptides in cultured S2 cells. Overexpression of Orbit/CLASP family proteins leads to the generation of microtubule bundles in the cytoplasm of cultured cells at interphase [38], [40]. (A) An S2 cell with overexpression of the full-length Orbit protein fused with mRFP tag (red). (B) An S2 cell with overexpression of the HRI region fused with mRFP tag (red). Anti-tubulin immunostaining (green). Scale bar5 µm.
(TIF)

Materials and Methods S1 (DOCX)
