# Supplementary figures and images for: Orbit/CLASP Is Required for Germline Cyst Formation through Its Developmental Control of Fusomes and Ring Canals in Drosophila Males
Source: PLoS One. 2013 Mar 8;8(3):e58220. doi: 10.1371/journal.pone.0058220 (PMC3592921; doi:10.1371/journal.pone.0058220)

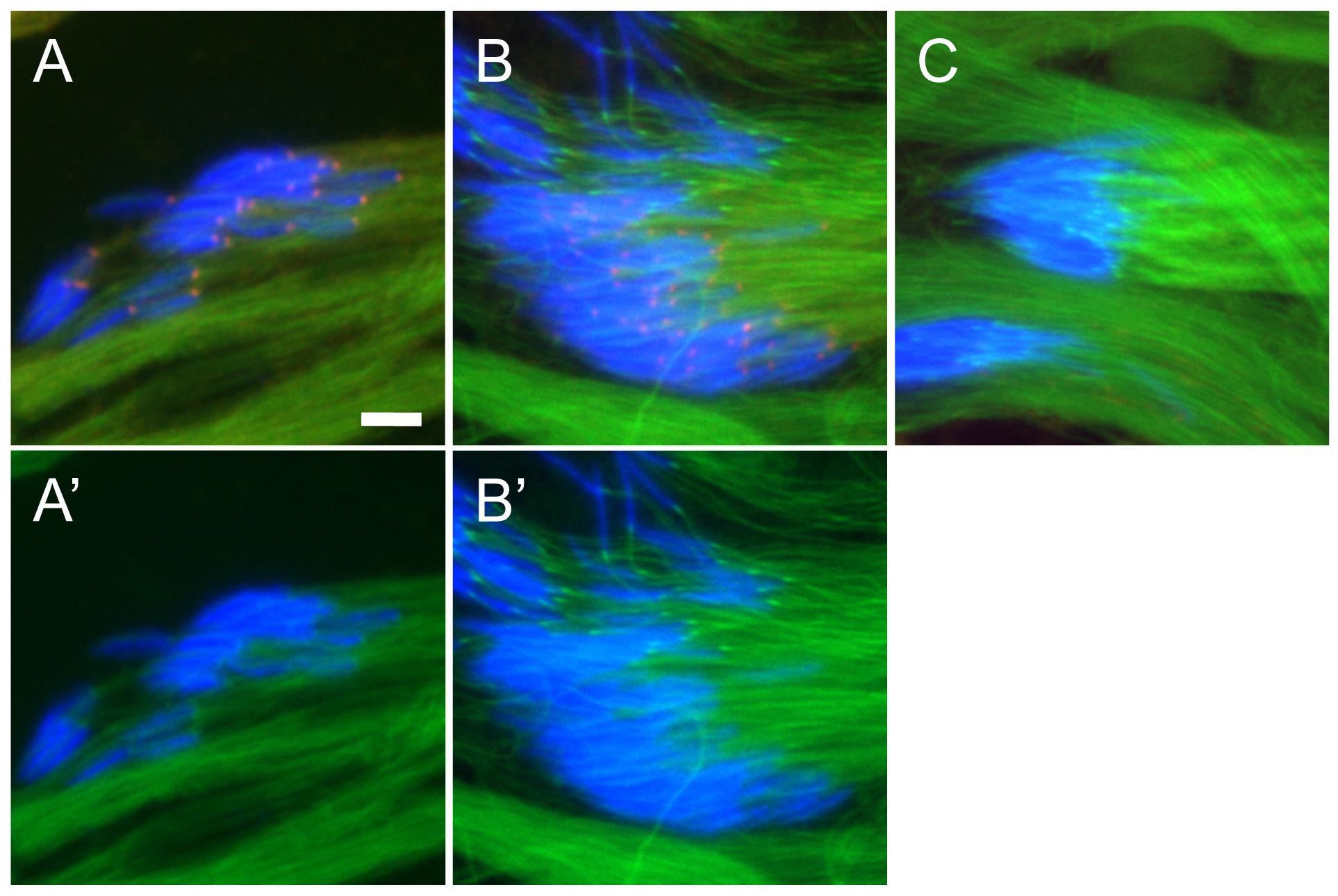

Supplement: Figure S1 — Immunostaining with anti-Orbit antibody appears temporary before basal body foci become prominent in elongated spermatids. (A–C) Immunostaining with anti-Orbit antibody (red), GFP-tubulin (green), and DNA staining (blue). (A) A cyst of elongated spermatids without distinct basal body foci. (A′) A merged image without red-channel presentation. The Orbit antibody recognizes a junction between the nucleus and axonema, in which basal bodies should be present. In a later elongated spermatid stage, the GFP-tubulin foci, corresponding to basal bodies, become conspicuous. The foci are not yet prominent in these spermatids. (B) An upper spermatid cyst in which spermatid individualization has been partially initiated is not stained with Orbit antibody. Immunostaining can be seen in the lower cyst, which contains earlier spermatids without basal body foci. (B′) A merged image without red-channel presentation. (C) In another two cysts of elongated spermatids, in which each spermatid appears to be tightly assembled, basal body foci are not stained with Orbit antibody. Scale bar = 10 µm. (TIF) [file pone.0058220.s001.tif]

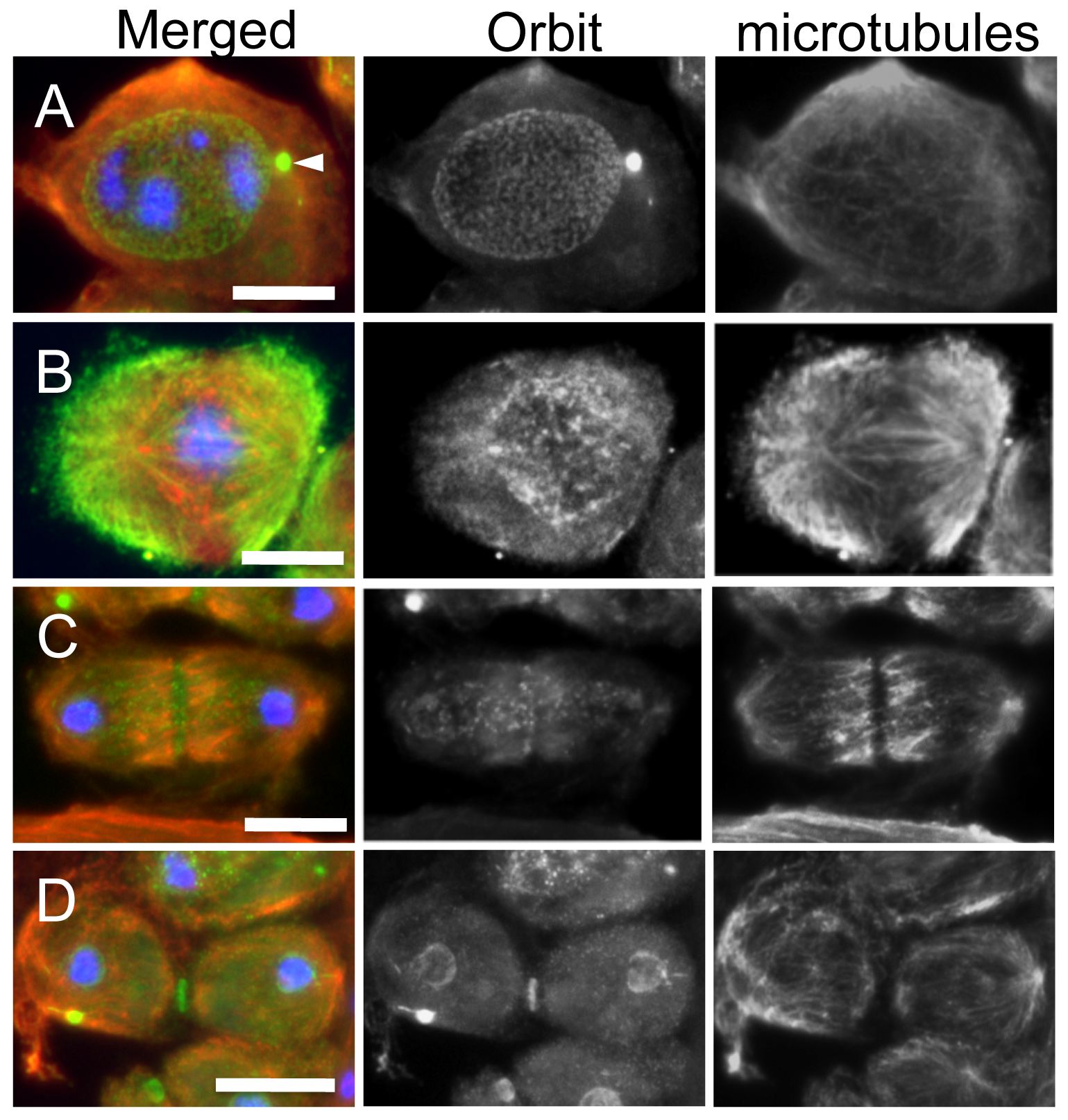

Supplement: Figure S2 — Cellular localization of Orbit with fluorescence tags in male meiotic cells. (A, C, D) Primary spermatocytes with expression of GFP-Orbit (green) at prophase (A), late anaphase (C), and cytokinesis stage (D). (B) Primary spermatocytes with mRFP-Orbit (red) at metaphase. Immunostaining with anti-tubulin antibody (red for A, C, D; green for B) was carried out to determine meiotic stages. Arrowhead in A indicates the remnants of the fusome, after its degradation before the initiation of meiosis. Scale bar = 10 µm. (TIF) [file pone.0058220.s002.tif]

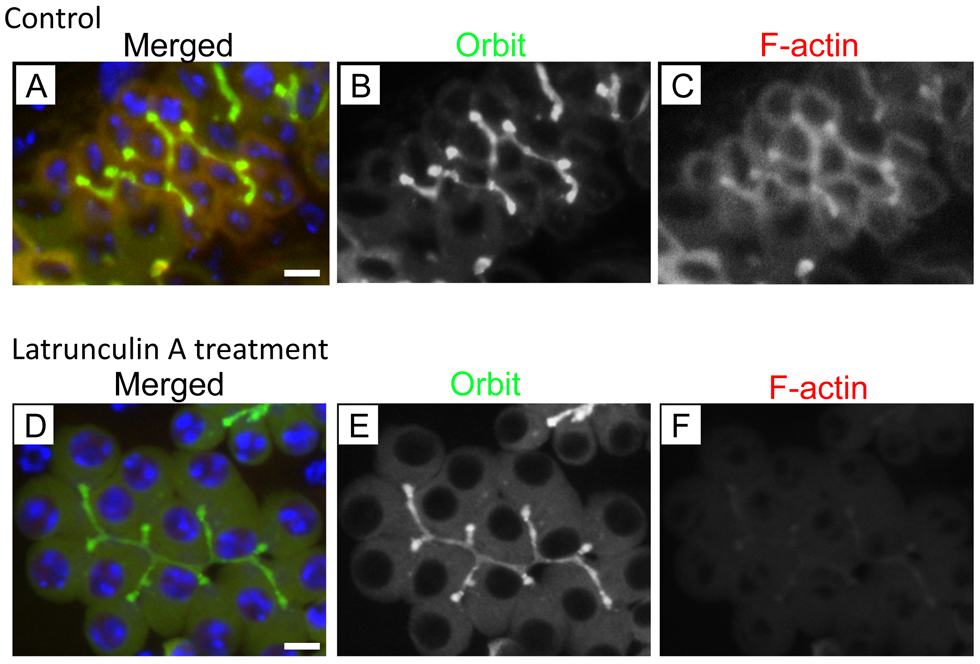

Supplement: Figure S3 — Inhibition of actin polymerization does not influence the maintenance of Orbit localization on fusomes. (A, D) RFP-actin (red), GFP-Orbit (green), and DNA (blue). (A–C) A control spermatocyte cyst in which F-actin and Orbit are components of extending fusomes. (D–F) F-actin depolymerization induced by treatment with latrunculin A influences neither Orbit localization on fusomes nor maintenance of the fusome structure. Scale bar = 10 µm. (TIF) [file pone.0058220.s003.tif]

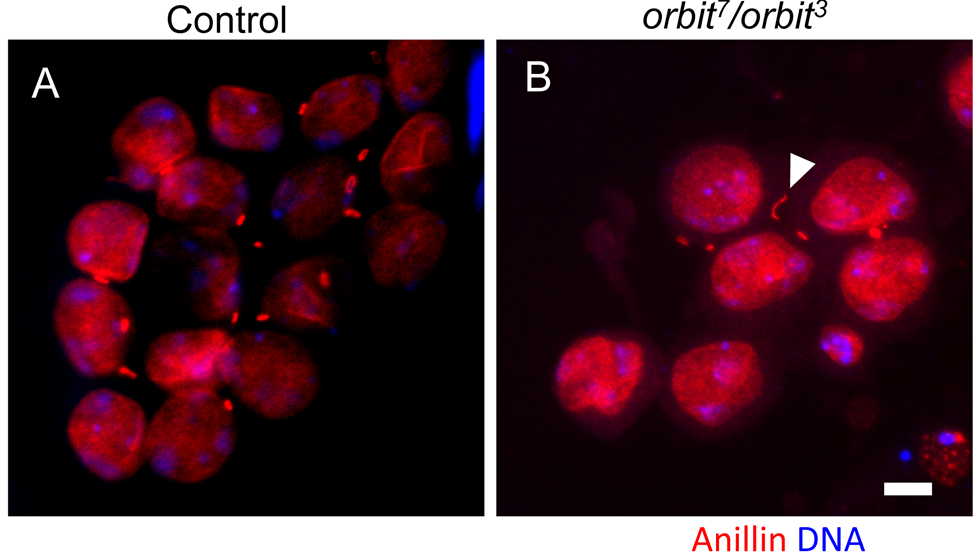

Supplement: Figure S4 — Abnormal organization and distribution of ring canals, visualized by immunostaining of anillin (a ring canal component), in a spermatocyte cyst from orbit7 mutant males. (A) In wild-type spermatocytes, normal (control) ring canals with constant diameter are distributed between every nucleus (blue). Anillin (red) is localized in nuclei and matured ring canals. (B) In spermatocyte cysts from orbit7 mutant males, ring canals with various diameters are observed. Two of the ring canals are distributed close to each other. Scale bar = 10 µm. (TIF) [file pone.0058220.s004.tif]

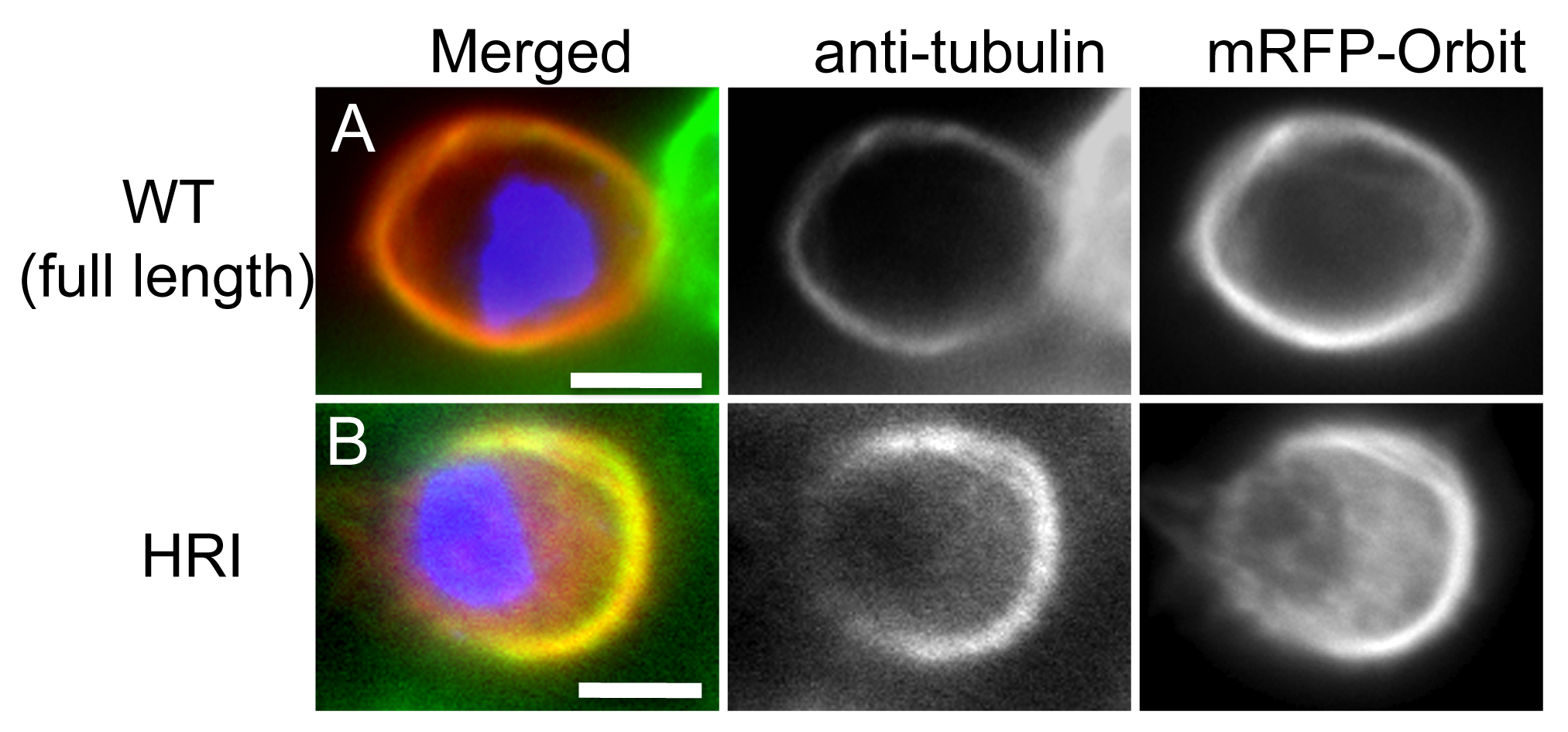

Supplement: Figure S5 — The full-length Orbit protein and HRI region are localized on microtubule bundles, induced by overexpression of the polypeptides in cultured S2 cells. Overexpression of Orbit/CLASP family proteins leads to the generation of microtubule bundles in the cytoplasm of cultured cells at interphase [38], [40]. (A) An S2 cell with overexpression of the full-length Orbit protein fused with mRFP tag (red). (B) An S2 cell with overexpression of the HRI region fused with mRFP tag (red). Anti-tubulin immunostaining (green). Scale bar5 µm. (TIF) [file pone.0058220.s005.tif]
