# Supplementary material for: Orbit/CLASP Is Required for Germline Cyst Formation through Its Developmental Control of Fusomes and Ring Canals in Drosophila Males
Source: PLoS One. 2013 Mar 8;8(3):e58220. doi: 10.1371/journal.pone.0058220 (PMC3592921; doi:10.1371/journal.pone.0058220)
Supplement: Materials and Methods S1 — (DOCX) [file pone.0058220.s006.docx]

**Materials and Methods**

Plasmids and transfection experiments

*Drosophila* S2 cells (1 × 10^6^), in a 5-cm diameter plate, were transfected with 1.0 μg of pUASp-mRFP-Orbit or pUASp-mRFP-HRI plasmids, and 1.0 μg of pActin-Gal4 (kindly provided by M. Yamaguchi), using Cellfectin (Invitrogen). The transfected S2 cells were transferred to cover glasses, fixed, and stained as described in Blagden et al. (2009).

Latrunculin A treatment of testis cells

Spermatocytes with co-expression of mRFP-actin and GFP-Orbit were dissected from testes, and incubated in a testis buffer containing 100 μM of latrunculin A (Molecular Probes), for 1 h at room temperature, before fixation.

**Fly stock**

The *orbit^3^* mutant was described previously [35].

**Reference**

Blagden SP, Gatt MK, Archambault V, Lada K, Ichihara K, Lilley KS, Inoue YH, Glover DM (2009) Drosophila Larp associates with poly(A)-binding protein and is required for male fertility and syncytial embryo development. Dev Biol 334: 186-197.
